# Identifying anal and cervical tumorigenesis-associated methylation signaling with machine learning methods

**DOI:** 10.3389/fonc.2022.998032

**Published:** 2022-09-29

**Authors:** Fangfang Jian, FeiMing Huang, Yu-Hang Zhang, Tao Huang, Yu-Dong Cai

**Affiliations:** ^1^ Department of Obstetrics & Gynecology, Ruijin Hospital, Shanghai Jiao Tong University School of Medicine, Shanghai, China; ^2^ School of Life Sciences, Shanghai University, Shanghai, China; ^3^ Channing Division of Network Medicine, Brigham and Women’s Hospital, Harvard Medical School, Boston, MA, United States; ^4^ Bio-Med Big Data Center, CAS Key Laboratory of Computational Biology, Shanghai Institute of Nutrition and Health, University of Chinese Academy of Sciences, Chinese Academy of Sciences, Shanghai, China; ^5^ CAS Key Laboratory of Tissue Microenvironment and Tumor, Shanghai Institute of Nutrition and Health, University of Chinese Academy of Sciences, Chinese Academy of Sciences, Shanghai, China

**Keywords:** cervical carcinoma, anal carcinoma, DNA methylation, machine learning, classification rule

## Abstract

Cervical and anal carcinoma are neoplastic diseases with various intraepithelial neoplasia stages. The underlying mechanisms for cancer initiation and progression have not been fully revealed. DNA methylation has been shown to be aberrantly regulated during tumorigenesis in anal and cervical carcinoma, revealing the important roles of DNA methylation signaling as a biomarker to distinguish cancer stages in clinics. In this research, several machine learning methods were used to analyze the methylation profiles on anal and cervical carcinoma samples, which were divided into three classes representing various stages of tumor progression. Advanced feature selection methods, including Boruta, LASSO, LightGBM, and MCFS, were used to select methylation features that are highly correlated with cancer progression. Some methylation probes including cg01550828 and its corresponding gene RNF168 have been reported to be associated with human papilloma virus-related anal cancer. As for biomarkers for cervical carcinoma, cg27012396 and its functional gene HDAC4 were confirmed to regulate the glycolysis and survival of hypoxic tumor cells in cervical carcinoma. Furthermore, we developed effective classifiers for identifying various tumor stages and derived classification rules that reflect the quantitative impact of methylation on tumorigenesis. The current study identified methylation signals associated with the development of cervical and anal carcinoma at qualitative and quantitative levels using advanced machine learning methods.

## 1 Introduction

Anal carcinoma is a malignant proliferative disease associated with anal abnormalities (1). With strong sex bias (females have higher mortality), more than 1000 people die of anal carcinoma per year in the United States (2, 3). The risk factors for anal carcinoma include aging, sex (more than two-thirds of patients are women), smoking, and most importantly human papilloma virus (HPV) infection (4, 5). Cervical carcinoma occurs in the cervix, which is located beneath the uterus and connects to the vagina (6). Smoking, immune suppression caused by human immunodeficiency virus (HIV) infection, and HPV infection are the major risk factors for cervical carcinoma (7). Both anal carcinoma and cervical carcinoma are malignant diseases associated with HPV infection. However, HPV cannot directly trigger the initiation and progression of such malignant diseases. The underlying mechanisms for HPV-mediated cancer initiation and progression have not been fully revealed. Therefore, for a long time, finding potential carcinogenic mechanisms associated with HPV infection and related biomarkers in anal and cervical carcinoma have been one of the major challenges in this field.

DNA methylation is a common biological process that regulates the activity of a DNA segment without changing the sequence (8, 9). It has been shown to be abnormally regulated during tumorigenesis in multiple cancer subtypes including anal and cervical carcinoma (10, 11). The demethylation of oncogenic genes and the methylation of tumor suppressors have been widely observed in cancers (8). In anal and cervical carcinoma, a genome-wide host methylation profiling under HIV infection revealed the potential associations between abnormal methylation status and anal and cervical carcinogenesis by monitoring the methylation alteration from normal to intraepithelial neoplasm and malignant tumorigenesis (12). Potential epigenetic markers to predict cancer risk and drive carcinogenesis around genes such as ASCL1, ATP10A, and CCDC81 have been identified. However, the quantitative association between biomarkers and disease risk has not been fully established.

In the present study, the methylation data, retrieved from Gene Expression Omnibus (GEO) database, on anal and cervical carcinoma samples was investigated. Three stages: normal control, intraepithelial neoplasia (also known as stage 0 of tumorigenesis reflecting the intermediate stage), and tumor, were included. To reveal the underlying biomarkers for distinguishing different stages, we applied multiple machine learning algorithms on the methylation data, which treated methylation as features. Some essential methylation sites were extracted, which can be latent biomarkers to distinguish different stages. Furthermore, some quantitative rules were also discovered for carcinogenesis monitoring, also indicating the different methylation patterns on various stages. Finally, some perfect classifiers were built to identify the stage of samples. All in all, this study provided a novel effective computational analysis for cancer biomarker recognition and progression monitoring on anal and cervical carcinoma.

## 2 Materials and methods

### 2.1 Data

The methylation profiling of 143 anal carcinoma samples and 28 cervical carcinoma samples was accessed from the GEO database under the accession number GSE186859 (12). The three different stages of cancer were involved in the 143 anal carcinoma samples: 9 normal samples, 13 anal intraepithelial neoplasia-3 (AIN3) samples, and 121 tumor samples. Similarly, the 28 cervical carcinoma samples contained 10 normal samples, 9 cervical intraepithelial neoplasia-3 (CIN3) samples, and 9 tumor samples. AIN3 or CIN3 is an intermediate state between normal and tumor. The 485,512 methylation probes were extracted for each anal and cervical carcinoma sample.

### 2.2 Boruta feature filtering

Because of the enormous number of original methylation features and limited methylations related to anal or cervical carcinomas, Boruta was employed for initial filtering (13–16).

Boruta is a random forest (RF)-based feature selection method for confirming whether variables in the classification are statistically superior to random variables. In a nutshell, Boruta analysis compares all variables to random variables, which are duplicates of the original variables by shuffling. RF is used to evaluate the importance of all variables, including actual and random variables. Actual variables that outperform the best random variables are labeled as confirmed, whereas those that do not outperform the best of the random variables are labeled as rejected. The above procedures are repeated numerous times, resulting in a binomial distribution for the binary outcome (confirmed or denied) of a series of *n* trials. The variables in the rejection region of the distribution were removed, whereas those in the acceptance region are preserved. To obtain the best classification accuracy, Boruta selects features that are strongly and weakly important, unlike wrapper techniques, which strive to discover a few powerfully relevant features.

The Boruta program used in this study was obtained at https://github.com/scikit-learn-contrib/boruta_py. It was performed on anal and cervical carcinoma samples using default parameters, respectively. Key methylation features for anal and cervical carcinomas were selected for further analysis, respectively.

### 2.3 Feature ranking algorithms

With Boruta, key methylation features for anal and cervical carcinomas can be obtained, respectively. However, they evidently provided different roles to depict anal or cervical carcinomas. Thus, further investigation was necessary. Here, three feature ranking algorithms: Monte Carlo feature selection (MCFS) (17), light gradient boosting machine (LightGBM) (18) and least absolute shrinkage and selection operator (LASSO) (19), followed to uncover the importance of features selected by Boruta. Their brief descriptions were as follows.

#### 2.3.1 Monte Carlo feature selection

In MCFS, the importance of features are determined according to their roles in multiple decision trees (DTs) (17). This method has been commonly used to process biological data (20–22). As part of the current study, *t* classification trees are built based on *m* randomly chosen methylation features and random division of training and test samples. Such procedures are executed *s* times. Consequently, *s*×*t* DTs are built, based on which a measurement, relative importance (RI), is computed for each feature. Such measurement is determined by how many times it has been selected in these *s*×*t* trees and how much it contributes to predicting the class of the *s*×*t* trees. It can be estimated as follows:


(1) $$RI_{g}=\;\sum_{\tau =1}^{st}{\left(wAcc\right)}^{u}\sum_{n_{g}\left(\tau \right)}IG\left(n_{g}\left(\tau \right)\right)\left(\frac{no.in\;n_{g}\left(\tau \right)}{no.in\;\tau }\right)v,$$

where *wAcc* is the weighted accuracy, *IG* (*n*_*g*_(*τ*)) is the information gain (IG) of node *n*_*g*_(*τ*) , (*no*.*in* *n*_*g*_(*τ*))  is the number of samples in node *n*_*g*_(*τ*) , and (*no*.*in* *τ*) is the sample sizes in the tree root. *u* and *v* are two settled positive integers.

The MCFS program was downloaded at https://home.ipipan.waw.pl/m.draminski/mcfs.html, which was executed using default parameters. According to the decreasing order of RI values, features were ranked in a list, named MCFS feature list.

#### 2.3.2 Light gradient boosting machine

The LightGBM algorithm uses a gradient boosting framework, and it is an improved version of the gradient boosting DT with the advantages of high efficiency, support for parallelism, and large-scale data processing (18). The total number of times each feature participated in the trees is used by LightGBM to evaluate the importance of features. The higher the frequency with which features are selected, the more important they are. Based on this criterion, features can be ranked in a list with the decreasing order of times.

In this study, we used the LightGBM program (https://lightgbm.readthedocs.io/en/latest/), implemented by Python. It was run under default parameters. The feature list generated by LightGBM was called LightGBM feature list.

#### 2.3.3 Least absolute shrinkage and selection operator

LASSO is a classic feature selection method (19). In this method, L1 paradigm is used to create a penalty function that selectively eliminates features by imposing a higher penalty on features with higher coefficients and more prediction errors, resulting in a model with fewer features and less overfitting. The coefficients of input features that do not contribute favorably to the prediction of a machine learning model are scaled down. As a result, the coefficients of the features are used to rank features in a list.

Here, the LASSO package collected in Scikit-learn (23) was used. Likewise, default parameters were used. For clear descriptions, the list yielded by LASSO was termed as LASSO feature list.

### 2.4 Incremental feature selection

Based on one feature ranking algorithm, a feature list can be obtained. However, which features are optimal for classification is still a problem. This study adopted incremental feature selection (IFS) method (24–28) to analyze the list and extract optimal features for a given classification algorithm. In this method, the feature list with *n* features is first divided into *n* feature subsets, with the number of features differing by 1 in turn. Subsequently, the feature subsets and target variables are fed into one classification algorithm to construct classifiers. Their classification performance is evaluated through 10-fold cross-validation (29). The optimal feature subset for one classification algorithm is defined as the subset of features with the highest classification performance and the classifier with the optimal feature subset is defined as the optimal classifier.

### 2.5 Synthetic minority oversampling technique

Two datasets for anal and cervical carcinomas, respectively, were investigated in this study. As mentioned in Section 2.1, the anal carcinoma dataset was imbalanced. In such dataset, tumor samples were about 13 times as many as normal samples. The classifiers directly built on such dataset would create bias. It was necessary to tackle such problem. In this study, synthetic minority oversampling technique (SMOTE) was adopted (30–32).

SMOTE is an oversampling method for dealing with imbalanced problems. It generates new samples for each minority class until the sizes of all classes are same. The samples of the minority class are synthesized by first selecting one sample to serve as a seed sample and then randomly selecting one of the *k* -nearest neighbors for linear combination. The synthesis formula is as follows:


(2)  s=x+β(x−y),

where *x* represents the feature vector of the seed sample, *y* represents the feature vector of its randomly selected neighbor, and *β* is a random value between 0 and 1. In this study, the SMOTE program downloaded from https://github.com/scikitlearn-contrib/imbalanced-learn was used. Default parameters were adopted to execute this program.

### 2.6 Classification algorithm

To execute IFS method, one classification algorithm was necessary. Two classic classification algorithms: RF (33) and DT (34), were attempted in this study as they are widely used in dealing with biological and medical problems (35–40). The below text gave the brief descriptions on these two algorithms.

RF is one of the most classic and powerful classification algorithms in machine learning. In fact, it is an ensemble algorithm containing multiple DTs. To construct each DT, samples are randomly selected, with replacement, from the original dataset and the selected sample number is equal to the number of samples in the original dataset. Furthermore, features are also randomly chosen from all features. RF integrates constructed DTs with majority voting. It is quite interesting that although DT is quite weak, RF is much more powerful and can avoid overfitting. Thus, it was adopted in this study to construct efficient classifiers.

Above-mentioned RF is generally much stronger than DT. However, it also loses the merits of its component DT. It is widely accepted that DT is a white-box algorithm, which means that its decision-making process is completely open. This makes it possible for us to understand the principle of DT. For the problems investigated in this study, DT can help us uncover essential methylation differences on three stages of anal and cervical carcinomas, thereby improving our comprehension on these two carcinomas. Generally, DT is a tree-like structure. There are two node types in this structure. One is branch node, which is in charge of determining which branch a test sample goes through down. The other is leaf node, which determines the class of the test sample reaching the leaf node. Besides, DT can also be represented by a set of classification rules. Each rule is generated by a path from the root to one leaf node. The investigation of these rules can uncover the different patterns of various stages of anal and cervical carcinomas.

To quickly implement DT and RF, related packages in Scikit-learn (23) were employed. These packages were executed using default parameters.

### 2.7 Performance evaluation

The weighted F1 was adopted to assess the overall performance of classifiers. To calculate such measurement, the F1 score on each class should be calculated first, which is defined as


(3) $$F1\;score_{i}=\frac{2\times TP_{i}}{2\times TP_{i}+FP_{i}+FN_{i}},$$

where *TP*_*i*_ is the true positive for the i-th class, *FP*_*i*_ and *FN*_*i*_ stand for the false positive and false negative for the i-th class. The weighted F1 is defined as the weighted mean of F1 scores on all classes. On the other hand, the direct mean of F1 scores on all classes defines another measurement, macro F1, which was also provided in this study.

Moreover, the accuracy (ACC) and Matthew correlation coefficients (MCC) (41) were also used in this study. ACC is the most classic measurement, which is defined as the proportion of correctly predicted samples. MCC is much more perfect than ACC when the class sizes are quite different. To compute the MCC, two binary matrices *X* and *Y* should be constructed in advance, where *X* and *Y* stores the true and predicted class of each sample, respectively. Then, MCC can be calculated by


(4) $$\text{MCC}=\frac{\text{cov}\left(\text{X},\text{Y}\right)}{\sqrt{\text{cov}\left(\text{X},\text{X}\right)\text{cov}\left(\text{Y},\text{Y}\right)}}$$

## 3 Results

**Figure 1:**
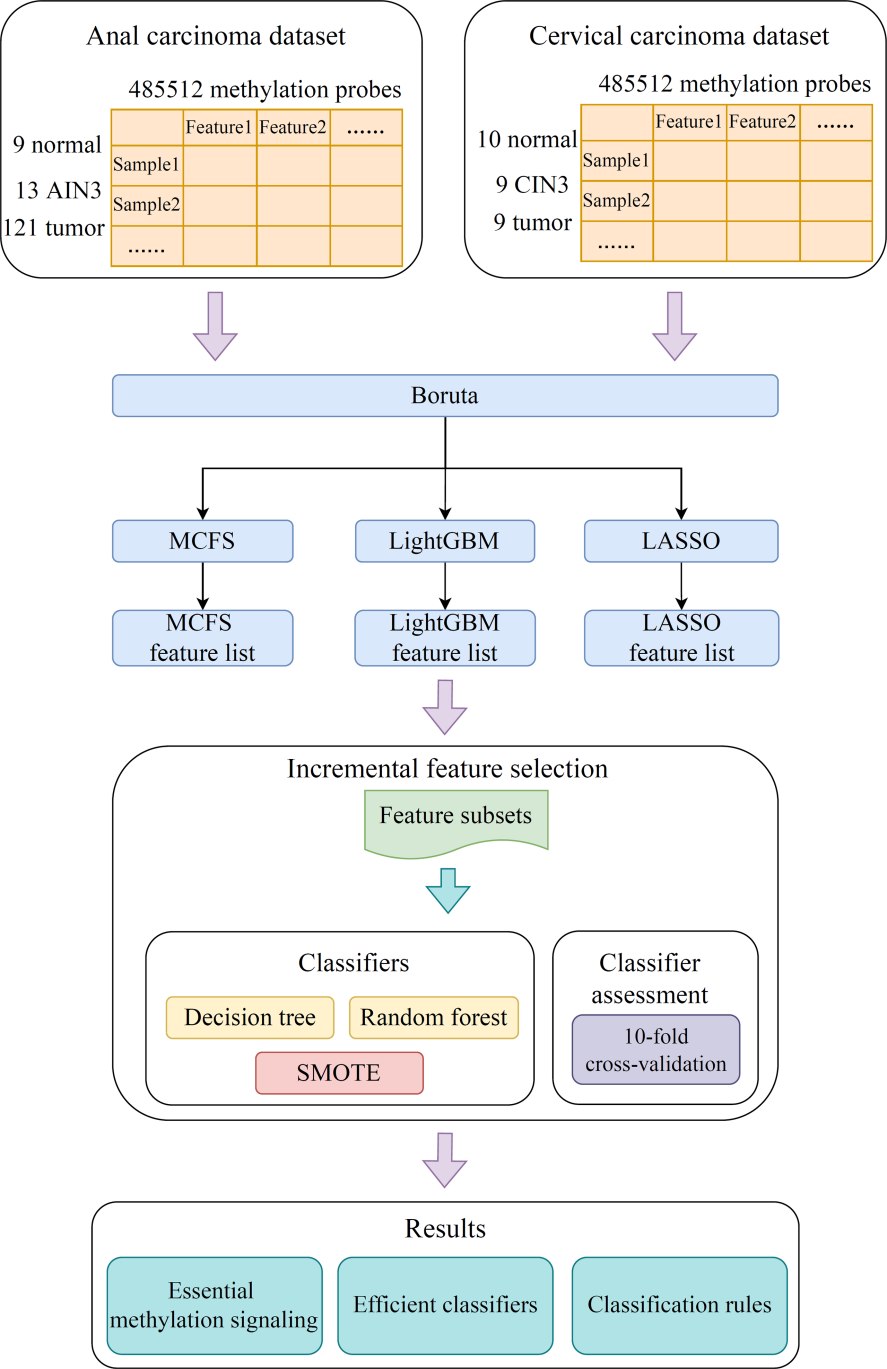
Flow chart of the entire analysis process. The 485,512 methylation probes in the anal or cervical carcinoma dataset are filtered by Boruta and ranked according to feature importance by using three feature ranking algorithms, namely, MCFS, LightGBM, and LASSO. Afterward, each of three feature lists is fed into the incremental feature selection (IFS) computational framework containing two efficient classification algorithms (decision tree, random forest) to extract essential methylations, construct efficient classifiers and classification rules.

In the present study, we developed a robust computational pipeline, which combined several machine learning algorithms. The entire procedures are illustrated in **Figure 1**. The detailed results were listed as below.

### 3.1 Results of Boruta and feature ranking algorithms

As lots of methylation features were used to represent each sample. Boruta was adopted for preliminary feature filtering. On anal carcinoma dataset, 571 methylation features were selected by Boruta, whereas 26 features were selected on the cervical carcinoma dataset. The selected features on two datasets are provided in **Supplementary Table S1**.

Subsequently, three feature ranking algorithms were used on both datasets to rank the filtered features by their importance. On each dataset, three feature lists were obtained, which are available in **Supplementary Table S1**. On the anal carcinoma dataset, three features were assigned RI values 0 by MCFS method. Thus, they were removed from the MCFS feature list. Furthermore, a biological analysis of how the top-ranked features affected the development of anal or cervical carcinomas would be given in Section 4.1.

### 3.2 Results of IFS method

Based on the three feature lists on each dataset, IFS method was executed using RF or DT as the classification algorithm. All possible feature subsets were constructed and RF or DT classifier was built on each of them, which was evaluated by 10-fold cross-validation. The cross-validation results are provided in **Supplementary Table S2**.

**Figure 2:**
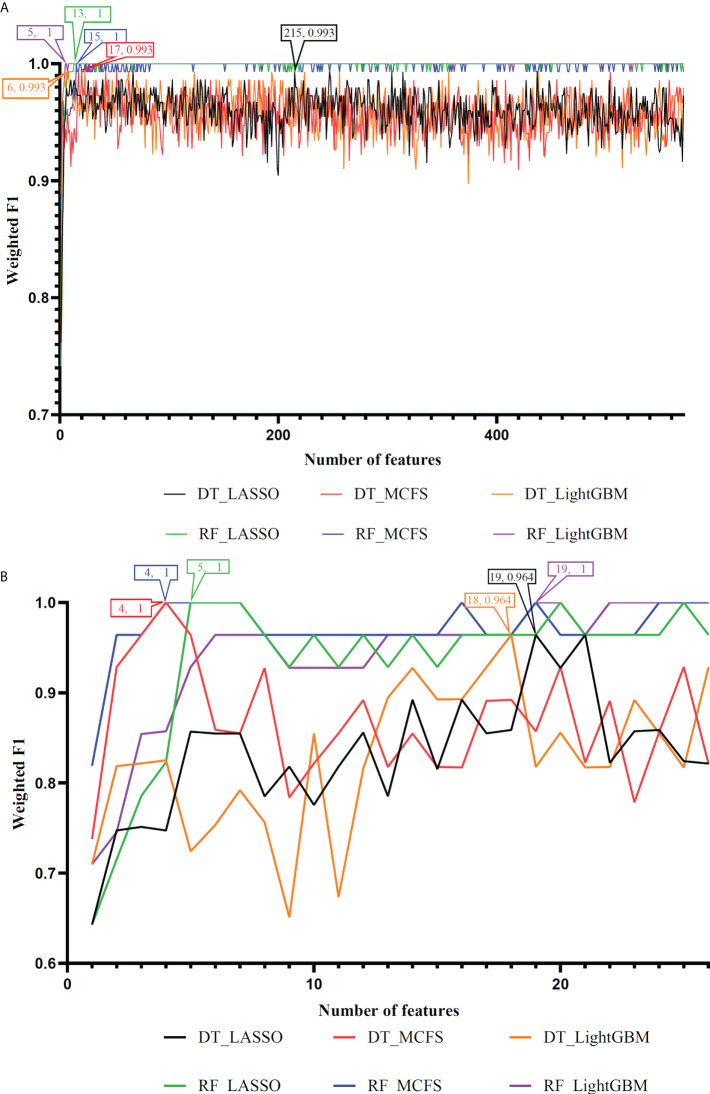
IFS curves to show the performance (weighted F1) of decision tree (DT) and random forest (RF) under different feature subsets in the anal and cervical carcinoma datasets. **(A)** IFS curves for the anal carcinoma dataset. **(B)** IFS curves for the cervical carcinoma dataset.

**Figure 3:**
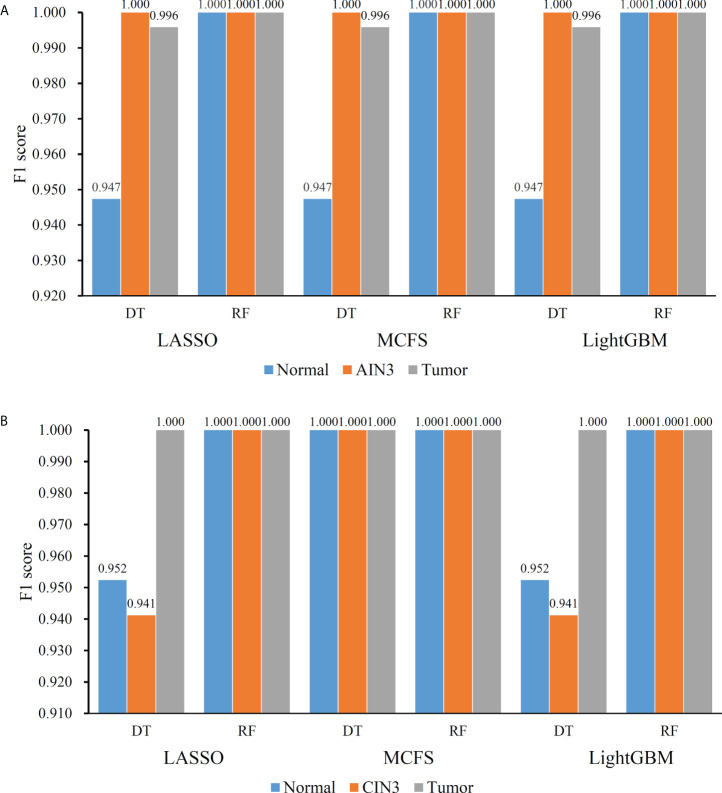
Performance of the optimal classifiers on three stages for anal or cervical carcinoma datasets. **(A)** Performance on the anal carcinoma dataset. **(B)** Performance on the cervical carcinoma dataset.

On the anal carcinoma dataset, the performance of classifiers, measured by weighted F1, was illustrated by six IFS curves, as shown in **Figure 2A**, in which weighed F1 was set as Y-axis and number of features was defined as X-axis. When DT was used in the IFS method, the highest weighted F1 values on the LASSO, MCFS and LightGBM feature lists, were all 0.993. Such performance was obtained by using top 215, 17 and 6 features in three feature lists, respectively. These features also comprised the optimal feature subsets for DT, on which three optimal DT classifiers were constructed. Their overall performance, measured by ACC, MCC and Macro F1, is listed in **Table 1**. Interestingly, their performance was same with ACC of 0.993, MCC of 0.975 and macro F1 of 0.981. Furthermore, the performance (F1 score) of these three DT optimal classifiers on three stages (normal, AIN3 and tumor) is shown in **Figure 3A**. The three classifiers also provided equal performance on three stages (0.947 on normal, 1.000 on AIN3 and 0.996 on tumor). Above results indicated the good performance of three optimal DT classifiers. As for the IFS results with RF, three curves were also plotted, as shown in **Figure 2A**. RF provided the perfect performance (weighted F1 = 1) on all three feature lists when top 13, 15 and 5 features in the LASSO, MCFS and LightGBM lists, respectively, were used. These features constituted the optimal feature subsets for RF on different lists. Accordingly, three optimal RF classifiers were set up with the optimal feature subsets. The ACC, MCC and macro F1 values of these classifiers are listed in **Table 1** and their performance on three classes is shown in **Figure 3A**. All measurements were equal to 1.000, also suggesting the perfect performance of three optimal RF classifiers.

**Table 1:** Performance of the optimal classifiers on anal carcinoma dataset.

Feature ranking algorithm	Classification algorithm	Number of features	ACC	MCC	Macro F1	Weighted F1
MCFS	DT	17	0.993	0.975	0.981	0.993
	RF	15	1.000	1.000	1.000	1.000
LightGBM	DT	6	0.993	0.975	0.981	0.993
	RF	5	1.000	1.000	1.000	1.000
LASSO	DT	215	0.993	0.975	0.981	0.993
	RF	13	1.000	1.000	1.000	1.000

On the cervical carcinoma dataset, the same IFS procedure was conducted. Three curves for DT and RF, respective, are plotted, as shown in **Figure 2B**. For IFS results with DT, the highest weighted F1 on the MCFS feature list was 1.000 and it was 0.964 on other two lists. The optimal feature subsets were constructed by picking up top 19, 4 and 18 features in the LASSO, MCFS and LightGBM lists, respectively. On these feature subsets, three optimal DT classifiers were set up. Their ACC, MCC and macro F1 values are listed in **Table 2**. Clearly, the optimal DT classifier on MCFS feature list provided perfect values on three measurements and the other two classifiers gave lower performance with ACC of 0.964, MCC of 0.948 and macro F1 of 0.965. Their performance (F1 score) on three stages (normal, CIN3 and tumor) is illustrated in **Figure 3B**. Again, the optimal DT classifier on MCFS feature list provided the perfect performance on all three stages and the other two classifiers yielded the same performance on three stages (0.952 on normal, 0.941 on CIN3 and 1.000 on tumor). Evidently, all three optimal DT classifiers generated perfect or nearly perfect performance. As for IFS results with RF, when top 5, 4, and 19 features in the LASSO, LightGBM and MCFS lists were adopted, RF produced perfect performance. The optimal feature subsets were constructed using these features and three optimal RF classifiers were built with them. These classifiers also provided perfect performance measured by other measurements (**Table 2** and **Figure 3B**).

**Table 2:** Performance of the optimal classifiers on cervical carcinoma dataset.

Feature ranking algorithm	Classification algorithm	Number of features	ACC	MCC	Macro F1	Weighted F1
MCFS	DT	4	1.000	1.000	1.000	1.000
	RF	4	1.000	1.000	1.000	1.000
LightGBM	DT	18	0.964	0.948	0.965	0.964
	RF	19	1.000	1.000	1.000	1.000
LASSO	DT	19	0.964	0.948	0.965	0.964
	RF	5	1.000	1.000	1.000	1.000

With the above IFS results, the optimal RF classifiers generally provided better performance than the optimal DT classifiers. All optimal RF classifiers yielded perfect performance, suggesting that they can be efficient tools to classify anal or cervical carcinoma samples.

### 3.3 Classification rules

One of the main purposes of this study was to depict the methylation patterns for two carcinomas on different stages. On anal carcinoma dataset, the top features in the LightGBM feature list were selected as they yielded the highest performance and they were least. The DT was applied on all anal carcinoma samples represented by these five features, yielding four rules, as listed in **Table 3**. Two rules were for identifying tumor samples and one rule was for predicting AIN3 and normal samples, respectively. Similarly, on the cervical carcinoma dataset, we selected the top four features in the MCFS feature list to construct the classification rules. Three rules were generated, as shown in **Table 4**. Each stage was assigned one rule. These rules would be discussed in detail in Section 4.2.

**Table 3:** Classification rules on anal carcinoma.

Index	Condition	Result
Rule 1	(cg01550828>0.0817) and (cg18954144>0.8291)	Tumor
Rule 2	cg01550828 ≤ 0.0817	AIN3
Rule 3	(cg01550828>0.0817) and (cg18954144 ≤ 0.8291) and (cg01550828>0.4363)	Normal
Rule 4	(cg01550828>0.0817) and (cg18954144 ≤ 0.8291) and (cg01550828 ≤ 0.4363)	Tumor

**Table 4:** Classification rules on cervical carcinoma.

Index	Condition	Result
Rule 1	cg10417457 ≤ 0.4173	Normal
Rule 2	(cg10417457>0.4173) and (cg02871554>0.6087)	CIN3
Rule 3	(cg10417457>0.4173) and (cg02871554 ≤ 0.6087)	Tumor

## 4 Discussion

By employing multiple machine learning algorithms, methylation datasets on anal and cervical carcinomas were deeply analyzed. Three feature lists, generated by three feature ranking algorithms, were obtained for each dataset. The methylation features with high ranks in three lists may be essential for two carcinomas, which can be novel methylation biomarkers associated with carcinoma progression from normal to precancerous lesions and from precancerous lesions to malignant cancer in anal and cervical carcinomas. Some of them were discussed in this section. Furthermore, some rules were set up for anal and cervical carcinomas, respectively. They were also discussed in the section.

### 4.1 Methylation biomarkers for anal and cervical carcinoma

The first methylation marker for anal carcinoma is cg23197559, near functional gene PTMA. According to recent publications, no direct reports confirm the association between PTMA and anal carcinoma. However, a recent study confirmed that in anal cancer and esophageal carcinoma, the methylation of a calcium-binding protein, S100A7, and PTMA has been shown to have specific methylation-mediated protein overexpression, validating the specific role of PTMA-related methylation alteration during anal carcinoma (42). The next probe cg07713411 is near gene MGA. MGA has been widely reported to be associated with tumor invasion (43) and progression (44). MGA has also been reported to contribute to MYC-mediated pathway in colorectal cancer cell lines. Considering the similarities between colorectal cancer and anal carcinoma, such gene regulated by our predicted methylation marker may also participate in the regulation of anal carcinoma, validating our prediction (44). The next probe cg25578064 regulates gene SFRS6, which is also a key driver gene for multiple gastrointestinal cancer subtypes (45), although it has no direct link with anal cancer. No functional genes were annotated around probe cg18954144 but the CpG site has been reported to be a typical signature for cancer overall survival (46), indicating that such probe may also be valuable for anal carcinoma monitoring. The final biomarker probe cg01550828 regulates a functional gene RNF168, encoding a ring finger protein. RNF168 has been selected as a candidate associated with HPV-related anal cancer (47), validating the efficacy and accuracy of our prediction.

As for biomarkers for cervical carcinoma, the first probe cg10417457 has been listed as a functional probe for cancer status monitoring according to a recent patent describing a systematic method to monitor cancer status established on 126 tumors (48). Therefore, such probe may also be functional to monitor cervical carcinoma. No reports associated with cg02871554 have been found. As for the probe cg27012396 near a functional gene HDAC4, various publications have confirmed that HDAC4 regulates the glycolysis and survival of hypoxic tumor cells in cervical carcinoma (49–51). Therefore, it is reasonable for us to predict that such probe may be a biomarker for cervical carcinoma. The next biomarker is cg05713971 near an effective gene called HERPUD1 Antineoplastic activity has been shown to be associated with gene HERPUD1 and further related to human cervical carcinoma according to a recent *in vitro* experiment (52). Such gene has also been detected to be regulated by functional microRNA miR-375 and further contributes to HPV-positive cervical cancer, validating our prediction (53).

### 4.2 Quantitative rules for anal and cervical carcinoma

For monitoring the status of anal carcinoma, three rules for recognizing three different clusters separately include the functional probes cg01550828 and cg18954144, both of which are associated with anal tumorigenesis as we have discussed above. According to our rules, a higher methylation level of cg01550828 and a lower methylation level of cg18954144 indicate a pathogenic status of anal carcinoma, consistent with previous studies (46, 47). Interestingly, we also identified lower methylation of cg01550828, associated with gene RNF168 as a biomarker for pathogenesis of intermediate status (precancerous lesions/intraepithelial neoplasia), providing a novel approach for predicting the precancerous lesion stage. As we have discussed above, all the top rules are established based on our qualitative biomarkers, indicating the consistency between different machine learning models and validating the efficacy and accuracy of our prediction.

For monitoring the status of cervical carcinoma, the top rules for normal control, precancerous lesion, and tumorigenesis prediction include the same group of features: cg10417457 and cg02871554. Although no direct association between cg02871554 and tumors has been recognized, cg10417457 has been validated to be an effective cancer-associated biomarker. Therefore, it is reasonable for our rules to summarize that a higher methylation of such probe may indicate a malignant change of cervical tissues. Further annotation on cg02871554 may be needed to explain its capacity for distinguishing precancerous lesions from malignant cancers.

## 5 Conclusion

In the present study, efficient feature selection algorithms, namely, Boruta, MCFS, LightGBM, and LASSO, were used to identify methylation signals associated with anal and cervical tumorigenesis. Subsequently, advanced machine learning algorithms were used to evaluate the performance of the filtered features for distinguishing different stages of anal or cervical carcinomas. Moreover, a DT was built to mine the classification rules for anal and cervical tumorigenesis. Taken together, this study provided a novel analysis to recognize key methylations for anal and cervical tumorigenesis qualitatively and quantitatively. The identified biomarkers and rules not only established an accurate and effective guideline for cancer differential diagnosis and progression stage monitoring, but also revealed potential mechanisms for the initiation and progression of anal and cervical tumorigenesis, indicating the specific roles of some methylations during the pathogenesis of these two diseases.

## Data availability statement

Publicly available datasets were analyzed in this study. This data can be found here: https://www.ncbi.nlm.nih.gov/geo/query/acc.cgi?acc=GSE186859.

## Author contributions

TH and Y-DC designed the study. FJ and FH performed the experiments. FJ, FH and Y-HZ analyzed the results. FJ and FH wrote the manuscript. All authors contributed to the article and approved the submitted version.

## Funding

This work was supported by the Strategic Priority Research Program of Chinese Academy of Sciences [XDA26040304, XDB38050200], the Fund of the Key Laboratory of Tissue Microenvironment and Tumor of Chinese Academy of Sciences [202002].

## Conflict of interest

The authors declare that the research was conducted in the absence of any commercial or financial relationships that could be construed as a potential conflict of interest

## Publisher’s note

All claims expressed in this article are solely those of the authors and do not necessarily represent those of their affiliated organizations, or those of the publisher, the editors and the reviewers. Any product that may be evaluated in this article, or claim that may be made by its manufacturer, is not guaranteed or endorsed by the publisher.

## Supplementary material

Supplementary Table 1 Feature lists yielded by MCFS, LightGBM, and LASSO on cervical and anal carcinoma datasets

Supplementary Table 2 Performance of DT and RF classifiers by applying IFS methods on feature lists yielded by MCFS, LightGBM, and LASSO on the cervical and anal carcinoma datasets.
